# The Contribution of Word-, Sentence-, and Discourse-Level Abilities on Writing Performance: A 3-Year Longitudinal Study

**DOI:** 10.3389/fpsyg.2021.668139

**Published:** 2021-08-03

**Authors:** Lénia Carvalhais, Teresa Limpo, Luísa Álvares Pereira

**Affiliations:** ^1^Portucalense Institute for Human Development (INPP), Department of Psychology and Education, Universidade Portucalense, Porto, Portugal; ^2^Faculty of Psychology and Education Sciences, University of Porto, Porto, Portugal; ^3^Department of Education and Psychology, University of Aveiro, Aveiro, Portugal; ^4^Centro de Linguística da Universidade Nova de Lisboa, Universidade Nova de Lisboa, Lisboa, Portugal

**Keywords:** writing performance, levels of language, grade level, longitudinal study, spelling, syntactic complexity measures, descriptive text

## Abstract

Writing is a foundational skill throughout school grades. This study analyzed the development of different levels of written language (word, sentence, and discourse) and explored the relationship between these levels and writing performance. About 95 Portuguese students from two cohorts—Grades 4–7 (*n* = 47) 6–9 (*n* = 48)—were asked to produce a descriptive text two times, with a 3-year interval. The produced texts were used to assess spelling, syntactic correctness and complexity, and descriptive discourse as well as text length and quality. The main results showed that there were improvements from Grades 4 to 7 and 6 to 9 in word- and sentence-level skills, along with increases in some dimensions of the descriptive discourse. Moreover, the older cohort performed better than the younger cohort in terms of spelling, syntactic complexity, and text quality, but not in terms of syntactic correctness, one dimension of the descriptive discourse, and text length. Regression analyses showed that writing performance was predicted by word and sentence levels in the younger cohort only, and by discourse-level variables in both cohorts. Overall, despite indicating a generalized growth in writing skills throughout schooling, this study also highlighted the areas that may need additional attention from teachers, mainly in terms of the descriptive features.

## Introduction

Writing is a complex skill (Dockrell et al., 2014). It requires the production of legible letters following conventional spellings, to produce words that are organized into sentences and form a coherent written text, expressing the writer's ideas (Abbott et al., 2010). Given the complexity of writing, research into the development of this ability throughout schooling is particularly relevant to understand the trajectories of learning and, based on these, to provide educational guidelines to foster writing skills. Much of extant research provides cross-sectional comparisons (Lerkkanen et al., 2004), and a few studies provide a longitudinal analysis of writing development (Berninger et al., 2010; Jagaiah et al., 2020), mainly with long gaps between the measurement points. These may help to better gauge the development of writing, given its long learning curve. This was the goal of the present study in which we examined the development of different levels of written language and their contribution to writing performance in two cohorts of Portuguese students (from Grades 4 to 7 and 6 to 9). These grades were chosen as they represent critical transitions for Portuguese students, from the first cycle of basic education (Grades 1–4) to the second one (Grades 5–6), and from the second cycle to the third cycle (Grades 7–9).

Levels of language, an analytic tool, is used to understand the complexity of oral and written language (Berninger and Garvey, 1982) based on the analysis of words, sentences, and discourse. In the word level, spelling could be defined as the ability to retrieve, assemble, and select orthographic symbols (Abbott and Berninger, 1993). Large-span cross-sectional studies found that spelling errors (an indicator of spelling skill) decreased throughout schooling, for example, from Grades 2 to 5 (Alves and Limpo, 2015), Grades 2 to 6 (Llaurado and Dockrell, 2020; Magalhães et al., 2020), and Grades 1 to 9 (Bahr et al., 2012). However, a few studies examined the type of errors produced, which can inform about the spelling difficulties of students in each language. Portuguese is a romance language with a simple syllabic structure and orthographic complexities and inconsistencies, classified as an intermediate depth orthography (Seymour et al., 2003; see more details about the Portuguese spelling system in Supplementary Material, section 1). Among several error categorization systems (Treiman et al., 2019), phonological, orthographic, and morphological assessment of spelling (POMAS) seems to be particularly useful, given its specificity of analysis and theoretical support. Grounded on the triple word form theory (Bahr et al., 2012), POMAS codes misspellings into three categories: phonological, orthographic, and morphological. Findings from POMAS revealed that from Grades 1 to 9 there was a decrease in phonological errors coupled with an increase in morphological ones, with most errors across grades being orthographic (Bahr et al., 2012). Despite not assessing morphological errors, Magalhães et al. (2020) found a similar pattern in Portuguese children from Grades 2, 4, and 6. The authors also found that stress marks errors—largely underexplored in Portuguese studies—were present equally in the assessed grades.

Writing a text also requires sentence-level abilities as children need to convert their ideas into sentences. Two key sentence-level measures are syntactic complexity and correctness (Dockrell et al., 2014). One of the most frequent measures of syntactic complexity is clause length (Jagaiah et al., 2020), which is the mean number of words per clause (Berman and Slobin, 1994). Syntactic correctness can be measured through the correctness of word sequences, defined as two contiguous syntactically and semantically acceptable writing units (Videen et al., 1982). A systematic review on Grades 1–12 concluded that syntactic complexity increased throughout schooling (Jagaiah et al., 2020). Similar evidence was found for syntactic correctness. In Grades 3–5, Dockrell et al. (2014) found that younger students produced significantly less correct word sequences than older ones. Likewise, similar findings were found by Malecki and Jewell (2003) showed that several indicators of syntactic correctness consistently increased from early elementary (Grades 1–2) to elementary grades (Grades 3–5), and from elementary to middle grades (Grades 6–8).

By serving specific communicative goals and functions, writing a text requires discourse-related knowledge concerning the structural features of each genre (Berman and Nir-sagiv, 2007; Graham et al., 2013; Dockrell et al., 2014). Like word and sentence levels, discourse-level abilities seem to increase throughout schooling, with students progressively producing texts with more and more genre-specific features. Tolchinsky (2019) found that descriptiveness (i.e., degree to which descriptive texts include the representative features of this genre) increased from Grades 1 to 4. Berman and Nir-sagiv (2007) found similar increases across grades in narrative and expository writing in older samples (Grades 4, 7, and 11 and University).

In addition to improvements in word, sentence, and discourse levels throughout schooling, research has shown that mastering these levels is important for writing performance, assessed in terms of the quality and amount of writing (Berninger, 2012). Both in primary and middle grades, a few studies found that better writing performance is predicted by (a) higher spelling skills [Grades 1–3 in Graham et al. (1997); Grades 3–6 in Abbott et al. (2010); and Grades 7–8 in Limpo et al. (2017)], (b) greater sentence-level abilities [Grades 2 and 3 in Arfé et al. (2016); Grades 3–5 and 5–7 in Beers and Nagy (2011); and Grades 7–8 in Limpo et al. (2017)]; and (c) more genre-related knowledge, including descriptive texts (Beers and Nagy, 2011; Tolchinsky, 2019). Providing stronger evidence on these links, meta-analyses showed that interventions promoting the writing levels of students improved the overall writing performance [Grades 1–6 in Graham et al. (2012) and Grades 4–12 in Graham and Perin (2007)].

### Present Study

As most findings surveyed above came from cross-sectional studies, it seems crucial to complement them with longitudinal findings to bring new inputs about writing development. To that end, the following research questions were addressed in a two-cohort sample of Portuguese students: Which are the developmental trajectories of word, sentence, and discourse levels of written language? Moreover, to which degree do these levels predict writing performance? Word, sentence, and discourse levels were measured through spelling, syntactic complexity/correctness, and descriptiveness, whereas writing performance was measured *via* text length and writing quality. The younger and older cohort were assessed at Grades 4 and 7 and 6 and 9, respectively. Based on the previous research, we expected a skill increase in all levels and an association between these and writing performance. We also hypothesized that the older cohort would show better writing skills than the younger cohort.

## Method

### Participants and Design

Participants were 101 students from Grades 4 to 9 and enrolled in a cluster of public schools located in urban middle-class neighborhoods from the Center of Portugal. Among these, six children were dropped from the analyses based on the following criteria: four had special education needs and two were identified as extreme outliers in one of the variables under analysis (viz., morphological misspellings per 100 words, which lied more than 3.0 times the interquartile range above the third quartile). All analyses were then based on the data from 95 students and were divided into two cohorts that were assessed twice (T1–T2), with a 3-year gap. The younger cohort was composed of 47 students in Grade 4 (51% girls) with an average age of 9.28 years at T1 (SD = 0.45). The older cohort included 48 students in Grade 6 (62% girls) with an average age of 11.35 years at T1 (SD = 0.53).

### Procedure

After the formal agreement from the principal of the school cluster, permission was given to contact the teachers of Grade 4 classes (a total of five) and Grade 6 classes (a total of four). After being explained about the goals and procedures of the study, including the possibility to withdraw at any moment, all teachers and students agreed to participate. In group, students were asked to produce a descriptive text in response to the prompt “Please describe your school,” which has been successfully used in previous research (e.g., Berninger et al., 2009; Dockrell et al., 2014). The full administration procedure lasted for 50 min. This is the typical duration of writing tasks in the participating schools, also used in prior studies (e.g., Llaurado and Dockrell, 2020). The exact same procedure was followed for both cohorts and testing moments. To potentiate the engagement of students, they were told that the best texts would be posted at the school webpage.

### Measures

Further details on the measures described below can be found in Supplementary Material, sections 2 and 3.

#### Word-Level Measures

Based on POMAS (Bahr et al., 2012), misspellings were counted separately by category: phonological errors, orthographic errors, morphological errors, stress marks, and illegible errors. However, the illegible errors were ignored as they were negligible (below 1%). This measure was re-scored by a second judge in the written products of 20% of the pupils at both T1 and T2. Reliability was good for all misspelling types, as indicated by the intraclass correlation coefficient (ICC) for single measures (>0.80).

#### Sentence-Level Measures

The sentence-level measures included the clause length and percentage of incorrect word sequences. Clause length was computed by averaging the number of words per clause, employing the computerized language analysis (CLAN) software (MacWhinney, 2000). The percentage of incorrect word sequences was calculated by examining the total number of incorrect sequences divided by the total number of sequences. Based on the scoring of 20% of the measures by a second judge, we concluded that reliability was high (ICC for individual measures >0.94).

#### Discourse-Level Measures

To measure descriptiveness (i.e., the presence of features typical of the descriptive text), we followed the taxonomy proposed by Adam (2001), including anchoring, aspectualization, relation, and subthematization categories. This taxonomy was used in previous studies, which relied on a dichotomous scale to indicate the presence or absence of the category [Grades 4–6 in Moura et al. (2015); Grade 3 in Pereira and Gonçalves (2017)]. Because our sample was older and we were concerned that this dichotomic coding would lead to ceiling effects, we added a new level indicating the presence and elaboration of information in each category. Thus, we used a three-point scale from 0 to 2, with the highest scores indicating a higher degree of discourse elaboration [for a similar coding scheme in argumentative texts, see Limpo and Alves (2013)]. Two independent judges scored these dimensions across all texts. Disagreements were solved through a discussion.

### Writing Performance

Two measures were used: text length and text quality. Text length was measured through the total number of words provided by CLAN. Text quality was assessed using a holistic scale ranging from 1 (*low quality*) to 7 (*high quality*), on creativity, coherence, syntax, and vocabulary (Alves et al., 2016). To avoid transcription biases on quality assessment, texts were previously typed, and misspellings were corrected (Berninger and Swanson, 1994). Two independent judges rated the text quality of all texts produced. Inter-reliability was high, as measured by the ICC for average measures, which was 0.91 at T1 and T2.

## Results

Excepting one measure, we confirmed that our study revealed no distributional problems, as the absolute values of these indexes of skewness and kurtosis did not exceed 3.0 and 10.0, respectively (Kline, 2005). We found a ceiling effect in the descriptive dimension of aspectualization in the younger cohort at T2. Thus, this variable was not included in the analyses. Descriptive statistics for variables are presented on Table 1.

**Table 1:** Means and SD for all variables by cohort and time.

	**Younger cohort**				**Older cohort**			
	**T1 (Grade 4)**		**T2 (Grade 7)**		**T1 (Grade 6)**		**T2 (Grade 9)**	
	***M***	***SD***	***M***	***SD***	***M***	***SD***	***M***	***SD***
**Word level (per 100 words)**
Phonological misspellings	0.97	1.35	0.50	0.79	0.64	0.89	0.17	0.34
Orthographic misspellings	1.77	1.45	0.98	0.99	1.11	1.42	0.40	0.55
Morphological misspellings	0.91	1.01	0.66	0.78	1.02	1.10	0.51	0.59
Stress mark misspellings	2.84	2.46	1.71	1.84	1.88	1.65	0.80	0.88
**Sentence level**
Incorrect word sequences (%)	6.59	3.15	4.00	2.63	5.89	2.94	4.72	3.38
Clause length	5.11	0.84	5.23	0.83	5.37	0.95	6.07	1.13
**Discourse level**
Anchoring	0.60	0.58	0.28	0.45	0.27	0.45	0.17	0.43
Sub thematization	1.11	0.73	1.60	0.58	1.29	0.62	1.69	0.47
Relationship	0.13	0.34	0.34	0.52	0.27	0.54	0.29	0.50
**Writing performance**
Text length	141.49	43.61	169.06	50.30	137.44	49.66	177.04	51.14
Text quality	3.20	1.14	4.67	1.18	3.90	1.32	5.04	1.07

### Cohort and Time Differences at the Word Level

To examine whether the type of misspellings varied across cohort and time, we conducted a 2 (cohort [younger, older]) ×2 (time [T1, T2]) × 4 (misspellings type [phonological, orthographic, morphological, stress mark]) ANOVA, with repeated measures on the two last factors. The results revealed a main effect of time, [*F*_(1, 279)_ = 64.30, *p* < 0.001, η${}_{\text{p}}^{2}$ = 0.41], a main effect of misspellings type, [*F*_(1, 279)_ = 37.67, *p* < 0.001, η${}_{\text{p}}^{2}$ = 0.39], and a main effect of cohort, [*F*_(1, 93)_ = 10.76, *p* < 0.001, η${}_{\text{p}}^{2}$ = 0.10]. Additionally, there were significant two-way interactions of misspellings type with both cohort, [*F*_(3, 279)_ = 4.86, *p* = 0.003, η${}_{\text{p}}^{2}$ = 0.05], and time, [*F*_(3, 279)_ = 5.97, *p* = 0.001, η${}_{\text{p}}^{2}$ = 0.06]. These were decomposed with simple-effect analyses followed up by pairwise comparisons with Bonferroni adjustments, for misspellings type × cohort and misspellings type × time. Overall, the results showed that stress mark errors were the most frequent and that, except morphological errors, the younger cohort produced more misspellings of all types than the older one (for complete results, see Supplementary Material, section 4).

### Cohort and Time Differences at the Sentence Level

To examine whether the percentage of incorrect word sequences and clause length varied across cohort and time, we conducted two 2 (cohort [younger, older]) × 2 (time [T1, T2]) ANOVAs. For incorrect word sequences, the results revealed a main effect of time, [*F*_(1, 93)_ = 24.21, *p* < 0.001, η${}_{\text{p}}^{2}$ = 0.21], with a decrease from T1 to T2. For clause length, the results showed a main effect of time, [*F*_(1, 93)_ = 9.14, *p* = 0.003, η${}_{\text{p}}^{2}$ = 0.09], a main effect of cohort, [*F*_(1, 93)_ = 15.85, *p* < 0.001, η${}_{\text{p}}^{2}$ = 0.15], and an interaction between the two, [*F*_(1, 93)_ = 4.56, *p* = 0.04, η${}_{\text{p}}^{2}$ = 0.05]. Simple-effect analyses showed that clause length increased across time only for the older cohort, [*F*_(1, 93)_ = 13.45, *p* < 0.001, η${}_{\text{p}}^{2}$ = 0.13], and that clause length differed between the cohorts at T2 only, [*F*_(1, 93)_ = 17.11, *p* < 0.001, η${}_{\text{p}}^{2}$ = 0.16]. The older cohort produced longer clauses than the younger one.

### Cohort and Time Differences at the Discourse Level

To examine whether descriptive dimensions varied across cohort and time, we conducted a 2 (cohort [younger, older]) × 2 (time [T1, T2]) × 3 (descriptive dimensions [anchoring, sub-thematization, relationship]) ANOVA, with repeated measures on the two last factors. The results revealed a main effect of time, [*F*_(1, 186)_ = 6.38, *p* = 0.01, η${}_{\text{p}}^{2}$ = 0.06] and a main effect of descriptive dimensions, [*F*_(2, 186)_ = 293.42, *p* < 0.001, η${}_{\text{p}}^{2}$ = 0.76]. In addition, we found significant two-way interactions of descriptive dimensions with cohort, [*F*_(2, 186)_ = 5.90, *p* = 0.003, η${}_{\text{p}}^{2}$ = 0.06], and with time, [*F*_(2, 186)_ = 22.25, *p* < 0.001, η${}_{\text{p}}^{2}$ = 0.19]. These were decomposed with simple effects analyses followed up by pairwise comparisons with Bonferroni adjustments, for descriptive dimensions × cohort and descriptive dimensions × time. In general, the findings showed different performances between dimensions, with higher results in sub-thematization, which increased from T1 to T2 in both cohorts (for complete results, see Supplementary Material, section 4).

### Cohort and Time Differences in Writing Performance

To examine whether text length and text quality varied across cohort and time, we conducted two 2 (cohort [younger, older]) × 2 (time [T1, T2]) ANOVAs. For text length, the results revealed a main effect of time, [*F*_(1, 93)_ = 30.63, *p* < 0.001, η${}_{\text{p}}^{2}$ = 0.25], with pupils writing longer texts at T2 than T1. For text quality, the results showed a main effect of cohort, [*F*_(1, 93)_ = 6.99, *p* = 0.01, η${}_{\text{p}}^{2}$ = 0.07], with the older cohort producing better texts than the younger one; and a main effect of time, [*F*_(1, 93)_ = 94.80, *p* < 0.001, η${}_{\text{p}}^{2}$ = 0.51], with pupils writing better texts at T2 than T1.

### Contribution of Written Language Levels to Writing Performance

Table 2 presents the bivariate correlations between all variables at T1 and T2 for both cohorts. In general, we found that (a) for the younger cohort, writing performance was associated with word and discourse variables at T1, but only with discourse variables at T2 and (b) for the older cohort, text length was associated with sentence-level variables and text quality with spelling-level, sentence-level, and discourse-level variables at T1, whereas at T2 only discourse level was related to writing performance.

**Table 2:** Bivariate correlations for all variables at T1 and T2 for the younger cohort (below the diagonal) and the older cohort (above the diagonal).

	**Time 1**											**Time 2**										
	**1**	**2**	**3**	**4**	**5**	**6**	**7**	**8**	**9**	**10**	**11**	**1**	**2**	**3**	**4**	**5**	**6**	**7**	**8**	**9**	**10**	**11**
**Time 1**
1. Phonological misspellings	-	0.05	0.07	**0.33**	0.24	**0.33**	−0.01	−0.06	−0.003	−0.14	−0.12	**0.32**	0.25	0.03	0.03	−0.04	−0.17	0.09	−0.12	−0.12	−0.10	0.12
2. Orthographic misspellings	**0.33**	-	−0.08	0.24	0.08	−0.07	0.01	−0.08	0.19	0.02	−0.15	0.03	0.22	−0.01	**0.46**	0.11	−0.09	−0.03	0.21	0.08	0.02	0.01
3. Morphological misspellings	0.06	0.23	-	0.19	0.16	0.05	0.04	0.16	0.23	0.03	0.12	**0.31**	0.19	0.21	0.16	**0.30**	−0.24	**−0.30**	−0.01	−0.01	−0.14	−0.17
4. Stress mark misspellings	**0.58**	**0.38**	**0.31**	-	0.11	0.05	−0.08	−0.04	0.06	−0.13	−0.11	**0.40**	**0.40**	0.10	**0.42**	−0.01	0.02	−0.16	−0.27	−0.05	0.05	−0.05
5. Incorrect word sequences	0.26	0.18	0.16	0.06	-	0.22	0.08	0.14	−0.04	0.13	0.11	−0.02	−0.11	0.08	0.06	0.24	0.06	−0.02	−0.14	0.20	−0.14	−0.09
6. Clause length	0.20	0.16	−0.01	−0.04	0.27	-	−0.03	0.16	−0.03	0.02	0.17	0.12	0.15	0.24	0.09	0.22	0.13	−0.07	0.06	0.17	0.01	0.17
7. Anchoring	−0.24	−0.01	0.07	−0.23	0.08	−0.11	-	0.17	−0.14	0.15	0.01	0.12	0.10	0.18	−0.02	−0.06	−0.04	−0.02	−0.10	−0.07	0.12	0.02
8. Sub thematization	**−0.33**	**−0.32**	−0.12	−0.19	**−0.40**	−0.11	0.26	-	0.14	**0.46**	**0.48**	−0.02	−0.05	−0.03	0.13	−0.001	0.06	0.13	0.03	−0.07	0.17	**0.30**
9. Relationship	0.10	0.20	−0.01	−0.22	0.15	0.23	0.05	−0.15	-	0.15	0.24	−0.13	−0.16	−0.02	0.00	0.16	−0.16	−0.11	−0.16	−0.06	0.02	−0.02
10. Text length	**−0.31**	−0.16	0.16	−0.29	−0.02	0.20	**0.41**	**0.40**	0.14	-	**0.78**	−0.17	−0.23	−0.03	−0.11	−0.01	−0.08	0.27	−0.04	0.22	0.27	**0.33**
11. Text quality	**−0.32**	−0.20	−0.13	−0.48	−0.17	−0.18	**0.49**	**0.43**	0.16	**0.60**	-	−0.14	−0.21	−0.06	−0.10	−0.04	−0.02	0.07	0.02	0.03	0.22	**0.32**
**Time 2**
1. Phonological misspellings	0.21	**0.35**	−0.11	0.23	0.21	0.14	−0.05	**−0.32**	0.24	−0.22	−0.16	-	**0.63**	**0.35**	0.27	0.07	−0.20	−0.11	−0.15	−0.02	−0.01	−0.02
2. Orthographic misspellings	**0.30**	**0.62**	0.29	**0.45**	0.15	−0.14	−0.05	**−0.37**	0.13	−0.15	−0.13	**0.44**	-	0.08	0.19	0.10	−0.06	−0.11	−0.09	0.02	−0.17	−0.13
3. Morphological misspellings	−0.12	0.16	−0.11	−0.01	0.10	**−0.30**	−0.04	−0.22	−0.14	−0.27	−0.19	0.29	**0.36**	-	**0.39**	**0.32**	0.13	0.05	0.04	0.02	0.14	−0.12
4. Stress mark misspellings	**0.31**	0.19	0.17	**0.56**	0.09	−0.21	0.002	−0.07	−0.04	−0.20	−0.28	0.15	**0.40**	0.24	-	0.10	0.04	−0.06	−0.002	−0.20	0.20	0.001
5. Incorrect word sequences	−0.05	**0.32**	0.27	0.24	0.26	−0.11	0.16	0.19	0.02	−0.08	−0.09	0.10	**0.45**	**0.34**	0.18	-	0.23	−0.15	**−0.32**	0.25	−0.20	−0.22
6. Clause length	−0.09	−0.04	−0.10	−0.07	−0.22	−0.15	−0.19	−0.04	0.04	0.04	0.22	0.05	0.11	0.16	0.04	0.10	-	−0.02	−0.03	0.17	0.09	−0.11
7. Anchoring	−0.03	−0.14	−0.02	0.04	0.20	−0.01	0.27	−0.03	0.19	0.11	0.10	−0.06	−0.11	**−0.32**	0.02	0.01	−0.09	-	0.16	−0.03	**0.38**	**0.42**
8. Sub thematization	0.21	−0.12	−0.03	−0.03	0.03	0.18	0.09	0.21	0.05	0.002	−0.06	−0.09	**−0.35**	**−0.32**	−0.02	−0.21	−0.10	0.02	-	0.21	**0.49**	0.28
9. Relationship	−0.22	−0.11	−0.07	−0.07	−0.16	−0.02	−0.11	0.19	−0.01	0.03	0.03	−0.14	−0.21	−0.26	−0.20	−0.02	−0.06	0.05	0.18	-	0.17	0.04
10. Text length	0.03	−0.11	−0.02	0.02	−0.06	0.004	**0.35**	0.26	0.06	0.26	0.27	−0.10	0.04	−0.10	0.24	**0.32**	**0.36**	0.09	0.19	0.08	-	**0.62**
11. Text quality	−0.08	−0.08	−0.07	−0.17	**−0.32**	−0.07	**0.30**	**0.33**	0.05	**0.38**	**0.46**	−0.20	−0.16	**0.35**	−0.08	−0.06	**0.31**	0.07	**0.38**	**0.38**	**0.56**	-

*Correlations above |0.30| are significant at an alpha level of 0.05 and signaled in bold*.

To test the contribution of the three levels of language to writing performance, we conducted a set of stepwise regression analyses to predict text length and quality at T1 and T2, separately by cohort (final model estimates are presented on Tables 3, 4, for text length and text quality, respectively). For predicting text length and quality at T1, we progressively introduced word-, sentence-, and discourse-level variables at T1 step-by-step. For predicting text length and quality at T2, we introduced text length and quality at T1 as a first step, followed by a step-by-step inclusion of writing levels at T2.

**Table 3:** Parameter estimates of the final regression models predicting text length at T1 and T2 for each cohort.

	**Younger cohort**					**Older cohort**				
	***B***	***SE***	***b***	***t***	***p***	***B***	***SE***	***b***	***t***	***p***
**PREDICTING T1 TEXT LENGTH**										
**Word-level predictors**
Phonological misspellings	−4.65	5.62	−0.14	−0.83	0.41	−5.85	8.93	−0.10	−0.66	0.52
Orthographic misspellings	−3.25	4.47	−0.11	−0.73	0.47	1.40	5.25	0.04	0.27	0.79
Morphological misspellings	10.22	5.97	0.24	1.71	0.10	−2.93	6.86	−0.07	−0.43	0.67
Stress mark misspellings	−1.32	3.34	−0.07	−0.40	0.70	−2.86	4.68	−0.10	−0.61	0.55
**Sentence-level predictors**
Incorrect word sequences	0.10	2.04	0.01	0.05	0.96	2.12	2.53	0.13	0.84	0.41
Clause length	14.35	7.11	0.28	2.02	0.05	−1.41	8.02	−0.03	−0.18	0.86
**Discourse-level predictors**
Anchoring	21.32	10.54	0.28	2.02	0.05	8.36	16.02	0.08	0.52	0.61
Sub thematization	18.09	9.04	0.30	2.00	0.05	33.64	12.00	0.42	2.80	0.01
Relationship	15.89	18.20	0.12	0.87	0.39	11.28	14.01	0.12	0.81	0.43
**PREDICTING T2 TEXT LENGTH**										
T1 Text length	0.32	0.15	0.28	2.15	0.04	0.23	0.14	0.23	1.71	0.10
**Word-level predictors**
Phonological misspellings	−1.58	9.03	−0.03	−0.18	0.86	37.90	26.29	0.26	1.44	0.16
Orthographic misspellings	−5.58	8.67	−0.11	−0.64	0.52	−21.91	14.90	−0.23	−1.47	0.15
Morphological misspellings	−10.31	10.17	−0.16	−1.01	0.32	−4.52	13.05	−0.05	−0.35	0.73
Stress mark misspellings	8.67	3.79	0.32	2.29	0.03	14.53	7.74	0.25	1.88	0.07
**Sentence-level predictors**
Incorrect word sequences	7.60	2.75	0.40	2.77	0.01	−1.23	2.22	−0.08	−0.55	0.58
Clause length	22.23	7.55	0.37	2.94	0.01	7.13	5.84	0.16	1.22	0.23
**Discourse-level predictors**
Anchoring	0.47	14.67	0.00	0.03	0.98	31.38	15.00	0.26	2.09	0.04
Sub thematization	18.65	11.99	0.21	1.56	0.13	47.20	15.29	0.43	3.09	<0.001
Relationship	5.68	12.45	0.06	0.46	0.65	8.78	14.32	0.09	0.61	0.54

**Table 4:** Parameter estimates of the final regression models predicting text quality at T1 and T2 for each cohort.

	**Younger cohort**					**Older cohort**				
	***B***	***SE***	***b***	***t***	***p***	***B***	***SE***	***b***	***t***	***p***
**PREDICTING T1 TEXT QUALITY**										
**Word-level predictors**
Phonological misspellings	0.10	0.14	0.12	0.74	0.47	−0.21	0.23	−0.14	−0.92	0.36
Orthographic misspellings	0.02	0.11	0.02	0.15	0.88	−0.13	0.13	−0.14	−0.99	0.33
Morphological misspellings	0.00	0.15	0.00	0.03	0.98	−0.01	0.18	−0.01	−0.05	0.96
Stress mark misspellings	−0.19	0.08	−0.40	−2.28	0.03	−0.03	0.12	−0.04	−0.28	0.78
**Sentence-level predictors**
Incorrect word sequences	−0.02	0.05	−0.07	−0.49	0.63	0.04	0.06	0.09	0.60	0.56
Clause length	−0.22	0.17	−0.16	−1.26	0.21	0.18	0.20	0.13	0.88	0.39
**Discourse-level predictors**
Anchoring	0.66	0.26	0.33	2.56	0.02	−0.10	0.41	−0.03	−0.24	0.81
Sub thematization	0.45	0.22	0.29	2.04	0.05	0.87	0.31	0.41	2.84	0.01
Relationship	0.43	0.45	0.13	0.97	0.34	0.52	0.36	0.21	1.46	0.15
**PREDICTING T2 TEXT QUALITY**										
T1 text quality	0.43	0.12	0.41	3.45	<0.001	0.23	0.11	0.29	2.15	0.04
**Word-level predictors**
Phonological misspellings	−0.12	0.19	−0.08	−0.66	0.51	0.77	0.61	0.25	1.26	0.22
Orthographic misspellings	0.10	0.18	0.08	0.54	0.60	−0.32	0.35	−0.16	−0.92	0.36
Morphological misspellings	−0.27	0.21	−0.18	−1.28	0.21	−0.44	0.30	−0.24	−1.43	0.16
Stress mark misspellings	0.07	0.08	0.11	0.88	0.38	0.15	0.18	0.12	0.81	0.42
**Sentence-level predictors**
Incorrect word sequences	0.02	0.06	0.05	0.37	0.72	−0.01	0.05	−0.02	−0.12	0.91
Clause length	0.40	0.16	0.28	2.46	0.02	−0.02	0.13	−0.02	−0.17	0.87
**Discourse-level predictors**
Anchoring	−0.06	0.31	−0.02	−0.20	0.84	0.98	0.34	0.39	2.92	0.01
Sub thematization	0.71	0.25	0.35	2.84	0.01	0.53	0.35	0.23	1.51	0.14
Relationship	0.70	0.26	0.31	2.70	0.01	0.08	0.32	0.04	0.24	0.81

#### Predicting T1 Text Length

For the younger cohort, Steps 1 and 2 did not reach statistical significance, but the inclusion of discourse-level variables increased the amount of variance explained in text length for both the younger, *R*_change_ = 0.19, [*F*_change(3, 37)_ = 4.09, *p* = 0.01]. The final model with all predictors explained 43% of the variance in text length at T1, [*F*_(9, 37)_ = 3.04, *p* = 0.01]. Significant and independent predictors were clause length (*b* = 0.28) and the descriptive dimensions of anchoring (*b* = 0.28) and sub-thematization (*b* = 0.30). The first steps and the final model were, however, not significant for the older cohort, *R*^2^ = 0.26, [*F*_(9, 38)_ = 1.52, *p* = 0.18].

#### Predicting T2 Text Length

For the younger cohort, Step 1 with text length at T1 and Step 2 with word-level predictors did not reach statistical significance. However, the inclusion of sentence-level predictors at Step 3 increased the amount of variance explained in text length, *R*${}_{\text{change}}^{2}$ = 0.26, [*F*_change(2, 39)_ = 8.70, *p* = 0.001]. When Step 4 was added, there was no significant increase. Together, text length at T1 and the three levels of written language at T2 explained 47% of the text length at T2, [*F*_(10, 36)_ = 3.19, *p* = 0.01]. Significant and independent predictors were T1 text length (*b* = 0.28), stress mark errors (*b* = 0.32), incorrect word sequences (*b* = 0.40), and clause length (*b* = 0.37). For the older cohort, there was no significant contribution of Steps 1–3, but the inclusion of descriptive dimensions significantly raised the amount of variance explained, *R*${}_{\text{change}}^{2}$ = 0.26, [*F*_change(3, 37)_ = 6.43, *p* = 0.001]. The final model explained 49% of the variance in T2 text length, [*F*_(10, 37)_ = 3.61, *p* = 0.02]. Significant and independent predictors were the descriptive dimensions of anchoring (*b* = 0.26) and sub-thematization (*b* = 0.43).

#### Predicting T1 Text Quality

For the younger cohort, Step 1 with word-level predictors made a significant contribution to text quality, *R*^2^ = 0.23, [*F*_(4, 42)_ = 3.14, *p* = 0.02]. The inclusion of sentence-level predictors did not increase the amount of variance explained, but the inclusion of discourse-level predictors did, *R*${}_{\text{change}}^{2}$ = 0.22, [*F*_change(3, 37)_ = 5.37, *p* = 0.004]. Together, the word-, sentence, and discourse-level predictors explained 50% of the variance in text quality at T1, [*F*_(9, 37)_ = 4.05, *p* = 0.001]. The variables with significant and independent contributions were the number of stress mark errors per 100 words (*b* = −0.40) and the descriptive dimensions of anchoring (*b* = 0.33) and sub-thematization (*b* = 0.29). For the older cohort, none of the first steps nor the final model reached statistical significance, *R*^2^ = 0.32, [*F*_(9, 38)_ = 1.99, *p* = 0.07].

#### Predicting T2 Text Quality

Step 1 of the analyses, including T1 text quality, proved significant for both the younger, *R*^2^ = 0.22, [*F*_(1, 45)_ = 12.35, *p* = 0.001], and older cohorts, *R*^2^ = 0.08, [*F*_(1, 46)_ = 5.25, *p* = 0.03]. Similar across the two cohorts, Steps 2 and 3 did not reach significance, but the inclusion of discourse-level predictors on Step 4 increased the amount of variance explained in text quality for both the younger, *R*${}_{\text{change}}^{2}$ = 0.20, [*F*_change(3, 36)_ = 5.74, *p* = 0.003], and older cohorts, *R*${}_{\text{change}}^{2}$ = 0.21, [*F*_change(3, 37)_ = 4.10, *p* = 0.01]. The final model for the younger cohort explained 58% of the variance in text quality, [*F*_(10, 36)_ = 4.96, *p* < 0.001]. Significant and independent predictors were text quality at T1 (*b* = 0.41), clause length (*b* = 0.28), and the descriptive dimensions of sub-thematization (*b* = 0.35) and relationship (*b* = 0.31). The final model for the older cohort explained 37% of the variance in text quality at T2, [*F*_(10, 37)_ = 2.22, *p* = 0.04]. Only text quality at T1 (*b* = 0.29) and the descriptive dimension of anchoring (*b* = 0.39) made a significant contribution.

## Discussion

The first goal of the present study was to trace the developmental path of writing at word, sentence, and discourse levels. The overall results indicated that older students showed better performance at word and sentence levels than younger students, but mixed findings were found for the discourse level.

In line with prior studies (Alves and Limpo, 2015; Llaurado and Dockrell, 2020; Magalhães et al., 2020), we found a general decrease in misspellings from T1 to T2 and more misspellings in the younger than the older cohort. This finding is not surprising as students in higher grades had more years of formal instruction and therefore had more spelling knowledge and writing experience. Moreover, it has been suggested that in free writing older students may be better at selecting words they know how to spell correctly (Graham and Santangelo, 2014). A misspelling analysis revealed four noteworthy findings. First, the pattern of older students producing less misspellings than younger ones was not observed for morphological errors. This finding aligns well with the proposal of Bahr et al. (2012), who suggested that more morphological errors may occur in older students due to the use of more complex vocabulary. This may require advanced morphological (derivational) knowledge that takes time to growth (Nagy et al., 2006; Berninger et al., 2010). In the future, POMAS could be used to examine the development of misspellings beyond Grade 9. Second, phonological misspellings decreased from T1 to T2 in both cohorts, being the least frequent type of misspelling in the older cohort. This is in line with previous research (Bahr et al., 2012) and suggests that sound-based spellings are learned in the earliest phases of spelling development (Treiman and Bourassa, 2000). Third, stress mark errors were the most frequent misspelling in both timepoints and cohorts, also corroborating past findings with younger Portuguese students (Magalhães et al., 2020). Stress mark errors indicate poor lexical knowledge of stress and difficulties in prosodic and orthographic mapping (Defior et al., 2012). From an applied standpoint, this means that spelling instruction is not being entirely successful in fostering this kind of knowledge. Finally, after stress mark errors, orthographic misspellings were the most predominant errors in Grades 4, 6, and 7. This is a common finding in the field (Bahr et al., 2012; Magalhães et al., 2020; Mesquita et al., 2020), suggesting that the Portuguese orthographic complexities and inconsistencies take several years to be mastered. Future research seems to be needed for developing evidence-based practices for improving orthographic knowledge beyond primary grades.

In line with previous meta-analytic findings (Jagaiah et al., 2020), the sentence-level results showed longer clauses in the older than the younger cohort. Moreover, we found stronger time-related increases in the ability of students to craft complex sentences in the older cohort. These results indicate that improvements in syntactic complexity are more salient in older writers, as it has been proposed by scholars in the field (Hunt, 1970; Berninger et al., 2011). This may be related to teacher practices, with a sentence-related explicit instruction, including vocabulary and sentence expansion exercises only at later stages when students are more familiarized with complex genres (Connors, 2000). Our results showed a growth in syntactic correctness from Grades 4 to 7 and 6 to 9, with better performances in the older than the younger cohort. Similar findings have been reported in the field (Malecki and Jewell, 2003; Dockrell et al., 2014), indicating that the ability to craft syntactically correct sentences progresses throughout the primary and middle school. The measure of incorrect word sequences seems particularly sensitive to gauge that progress, including in older students (Espin et al., 2000; Weissenburger and Espin, 2005).

The analyses examining cohort and grade differences in the discourse level revealed three main findings. First, the younger cohort performed better than the older one in terms of anchoring. This unexpected finding may be related to the Portuguese curricula, which, in the initial grades, emphasize the use of titles and introductory sentences to contextualize the theme to readers (Buescu et al., 2015). Though *vivid* in late primary and early middle grades, these recommendations may be lost over the years. Second, we observed the anticipated increase from T1 to T2 in the ability of students to connect the prompt with other topics, with older students performing better than younger ones. Tolchinsky (2019) already suggested that older students tend to provide elaborated descriptions of topic-related aspects, whereas younger ones usually present lists of attributes, with a few efforts to articulate content. This progressive increase in ideas elaboration over time is common to other genres (Berman and Nir-sagiv, 2007; Beers and Nagy, 2009) and may be linked to progressive mastery of writing of students. Third, relationship was the most absent descriptive feature (either alone in Grade 4, or together with anchoring in Grades 6, 7, and 9). Clearly, ability of the students to relate and compare concepts was poor in all grades assessed, which is alarming, given the importance of this feature. Descriptive texts should establish links between sub-topics through comparisons or metaphors, which allow readers to form a picture in their minds (Adam, 2001). More research is needed to understand which factors underlie this poor performance and which strategies may be used to foster it.

The second goal of this study was to examine the contribution of word, sentence, and discourse levels to writing performance. However, considering the participants/predictors ratio, some caution is needed when interpreting these findings, which should be replicated in future studies with larger samples. Concerning word-level predictors, we found that more stress mark errors were associated with poorer texts at T1. This finding aligns with those of Magalhães et al. (2020) showing that in Grade 4, stress mark errors were reliable predictors of text quality. Regarding sentence-level predictors, we found the overall contribution of syntactic complexity to the amount and quality of students writing, confirming the importance of producing complex and good sentences to perform well in writing (Beers and Nagy, 2011; Limpo et al., 2017). Interestingly, the contribution of word- and sentence-level predictors to writing performance was only observed in the younger cohort, indicating that once students master a writing level, its role in writing performance diminishes (Graham, 2006). A striking finding involving both word- and sentence-level predictors at T2 in the younger cohort was that more stress mark errors, and more incorrect word sequences were associated with longer texts. Although this finding may be an artifact of the current study, it may also hint that by ignoring some aspects of writing, such as word stress and sentence correctness, students may be able to write more. Additional research is needed to replicate and explore these results.

Discourse-level variables were the most salient predictors of writing performance in both cohorts, confirming that the amount and quality of the writing of students is heavily dependent on their ability to follow genre-specific structures (Graham, 2006). This means that a powerful way to increase writing performance in a given genre is to improve student's knowledge about its underlying. Previous meta-analyses on the best methods to develop writing are in line with this conclusion (Graham and Perin, 2007; Graham et al., 2012). It should, however, be noted that our findings showed that not all descriptive categories contributed equally to writing performance. The more relevant category seems to be subthematization, that is, the ability of students to elaborate on the content. Experimental research is needed to examine the degree to which teaching of each of the descriptive categories results in better writing performance.

### Implications for Applied Settings

The findings of the current study provide relevant hints for practice. Despite the general growth in writing, there seems to be room for improvement. In younger students, teachers may need to provide additional instruction in terms of syntactic complexity, whereas in older students, they may need to focus on stress mark and orthographic knowledge as well as on descriptive features, mainly, anchoring.

## Conclusion

In sum, this study showed the overall growth in word and sentence levels and an increase in the discourse level only for sub-thematization. Moreover, whereas word and sentence level predicted writing performance only in the younger cohort, the discourse level was a relevant predictor in both cohorts. By helping us to understand the long-term curve of writing development, these findings provide hints for researchers to develop evidence-based practices tailored to the writing needs of students.

## Data Availability Statement

The raw data supporting the conclusions of this article will be made available by the authors, without undue reservation.

## Ethics Statement

Ethical review and approval was not required for the study on human participants in accordance with the local legislation and institutional requirements. Written informed consent from the participants' legal guardian/next of kin was not required to participate in this study in accordance with the national legislation and the institutional requirements.

## Author Contributions

LP was responsible for initial study design and data collection. LC coordinated data coding and the manuscript preparation. TL analyzed the data. All authors contributed to the design and conceptualization of the study, literature review, discussion, and wrote and reviewed the manuscript and approved its final version.

## Conflict of Interest

The authors declare that the research was conducted in the absence of any commercial or financial relationships that could be construed as a potential conflict of interest.

## Publisher's Note

All claims expressed in this article are solely those of the authors and do not necessarily represent those of their affiliated organizations, or those of the publisher, the editors and the reviewers. Any product that may be evaluated in this article, or claim that may be made by its manufacturer, is not guaranteed or endorsed by the publisher.
