# Supplementary material for: The Contribution of Word-, Sentence-, and Discourse-Level Abilities on Writing Performance: A 3-Year Longitudinal Study
Source: Front Psychol. 2021 Aug 3;12:668139. doi: 10.3389/fpsyg.2021.668139 (PMC8368977; doi:10.3389/fpsyg.2021.668139)
Supplement: Supplementary file 1 [file Data_Sheet_1.docx]

Supplementary Material

# Characterization of the Portuguese spelling system

Portuguese is a Romance language from the Indo-European family, that is the native language of more than 220 million people across more than 10 countries (Eberhand et al., 2019; Mesquita et al., 2020). Concerning spelling and the relation of sound-to-print correspondences, Portuguese orthography has 37 phonemes to 67 simple and complex graphemes (Gomes, 2001), with some phonemes with a univocal phoneme correspondence as, for example, the grapheme ‹t› that corresponds to one phoneme /t/, as ‹tela› *screen*. In other cases, there is no univocal correspondence between phonemes and graphemes and one phoneme could have two, three or four correspondences (Moutinho, 2000), that could be explicitly thought by rules (e.g., before /p/ and /b/ use grapheme ‹m›) or inconsistent (e.g., the phoneme /ʃ/ that could be represented by the graphemes ‹ch›, ‹x›, ‹s› or ‹z›, with no specific rule to determine the grapheme that should be selected by writers). Portuguese language also includes the diacritics, different stress marks /´/, /^/ and /~/ that indicate if the vowel is open or closed. In term of syllable structure, Portuguese words are mainly composed by a Consonant-Vowel (CV) structure, but there are also words composed by CCV or CCVC. At morphological level, Portuguese language contains lexical morphemes as well as grammatical morphemes (e.g., gender’s variation represented by -o/-a or the number represented by -s; see Cunha & Cintra, 2014).

# Explanation and examples of spelling errors categories

We calculated the percentage of misspellings by dividing the total number of errors per total number of words, and then multiplying by 100. Misspellings were analyzed by each linguistic category: phonological errors: when spelling did not present the correct phonological representation of sounds (e.g., sounds substitution ‹escala› instead of ‹escola› school); orthographic errors: include difficulties in represent specific patterns of Portuguese language, even the word presents the correct phonological feature (e.g., ‹fasso› instead of ‹faço› to do); morphological errors: morphological awareness includes both inflectional and derivational relations as well as compounding (e.g. ‹meio dia› instead of ‹meio-dia› noon or ‹chama-se› instead of ‹chamasse› to call); stress marks (diacritics are used to convey word stress and in the Portuguese language, the three diacritics mark stress indicate whether the vowel is open or closed as, for example, ‹fútebol› instead of ‹futebol› football ; and illegible errors (e.g., when is not possible to identify the written word).

# Explanation and examples of discourse-level categories and writing performance

Students’ descriptive texts were analyzed based on Adam’s theory (2001), who proposes that a descriptive text could be constructed using four dimensions. The anchoring results in the theme-title or an initial sentence presenting the main idea of the text (e.g., the title “My school” or an introductory sentence as “Today I will present to you, my school.”). Aspectualization consists of exposition of different aspects of the object or reality or describe parts of the object (e.g., the school rooms, the color of the windows, the daily routines). Relation comprises the establishment of comparisons or metaphors (e.g., “My school is like a sunflower.”). The sub thematization procedure is the operation that consists in linking one sequence in the other, with more specific details or functions for the aspects already presented (e.g., “My school has a playground where is possible to play football, gymnastic and walk with my friends.”). This sub thematization could be focused in personal or physical aspects or both as a hybrid description (e.g., “At school we have excellent staff because they help us in everything we need: they guide us, clean the rooms and the playgrounds and when we get hurt, they help us.”). Each element was awarded by two independent judges that started initially with a discussion of different procedures, using texts as anchors, for the construction of a punctuation table.

Following the four procedures proposed by Adam (2001), this task was scored with 8 points in total; 2 points maximum for each procedure. As an example, in the anchoring procedure, if the student presented a text without any title or introduction, the punctuation was zero; if there was just a title, the text was scored as 1; if there was a title and an initial sentence describing the theme of the text, judges scored two. For all the other descriptive text construction procedures, the same procedure was followed. An initial scoring of 10 texts were discussed by the two judges and all the other texts were recoded to avoid cohort or time (i.e., if the text resulted in the T1 or T2 production) scoring influence. A final interrater reliability was obtained when the two judges discussed each punctuation, and a full agreement was achieved.

At writing performance, text quality was assessed using a holistic scale ranging (e.g., Limpo & Alves, 2013) from 1 (low quality) to 7 (high quality), on creativity (i.e., novelty and relevance of presented ideas), coherence (i.e., connection and organization of the ideas presented in the text), syntax (i.e., sentence construction and correctness) and vocabulary (i.e., proper use of words, semantic diversity) (Alves et al., 2016).

# Results

**Cohort and Time Differences at Word Level**

**Misspellings type x Cohort.** The number of misspellings by 100 words varied across type for the younger cohort, *F*(3, 91) = 23.59, *p* < .001, η^2^_p_ = 0.44, as well as for the older cohort, *F*(3, 91) = 9.22, *p* < .001, η^2^_p_ = 0.23. In the younger cohort, the most frequent misspellings were stress mark errors (*p*s < .01), followed by orthographic misspellings. Phonological and morphological misspellings (produced at the same extent) were the least frequent errors (*p*s < .002). In the older cohort, the most frequent misspellings were stress mark errors (*p*s < .05), followed by morphological and orthographic misspellings (produced at the same extent). Phonological errors were the least frequent (*p*s < .04), though produced at the same extent as orthographic errors (*p* = .07). Additionally, phonological misspellings, *F*(1, 93) = 5.32, *p* = .23, η^2^_p_ = 0.05, orthographic misspellings, *F*(1, 93) = 9.70, *p* = .002, η^2^_p_ = 0.09, and stress mark misspellings, *F*(1, 93) = 8.67, *p* = .004, η^2^_p_ = 0.09 (but not morphological errors) were higher in the younger than the older cohort.

**Misspellings type x Time.** The number of misspellings by 100 words varied across type for T1, *F*(3, 91) = 25.00, *p* < .001, η^2^_p_ = 0.45, as well as for T2, *F*(3, 91) = 14.75, *p* < .001, η^2^_p_ = 0.33. Stress mark errors were the most frequent ones at T1 and T2 (*p*s < .001). At T1, the second most frequent misspellings were orthographical, which were higher than both phonological and morphological misspellings (*p*s < .05). At T2, the second most frequent misspellings were orthographical and morphological, which were higher than phonological misspellings (*p*s < .02). Moreover, phonological misspellings, *F*(1, 93) = 15.44, *p* < .001, η^2^_p_ = 0.14, orthographic misspellings, *F*(1, 93) = 32.09, *p* < .001, η^2^_p_ = 0.26, morphological misspellings, *F*(1, 93) = 9.01, *p* = .003, η^2^_p_ = 0.09, and stress mark errors, *F*(1, 93) = 15.44, *p* < .001, η^2^_p_ = 0.14 decreased from T1 to T2.

**Cohort and Time Differences at Discourse Level**

**Descriptive dimensions x Cohort.** Performance in descriptive dimensions varied among them in the younger cohort, *F*(2, 92) = 113.27, *p* < .001, η^2^_p_ = 0.71, as well as in the older cohort, *F*(2, 92) = 167.67, *p* < .001, η^2^_p_ = 0.79. In the younger cohort, the three descriptive dimensions differ among each other, with better performance on sub thematization, then on anchoring, and finally on relationship (*p*s < .02). Similar findings were observed for the older cohort, with the exception that performance was similar in the anchoring and relationship dimensions. Differences between cohorts were only found for the anchoring dimension, *F*(1, 93) = 8.51, *p* = .004, η^2^_p_ = 0.08, with the younger cohort showing better performance than the older one.

**Descriptive dimensions x Time.** Performance in descriptive dimensions varied among them at T1, *F*(2, 92) = 78.36, *p* < .001, η^2^_p_ = 0.63, and T2, *F*(2, 92) = 277.46, *p* < .001, η^2^_p_ = 0.86. For T1, the three descriptive dimensions differ among each other, with better performance on sub thematization, then on anchoring, and finally on relationship (*p*s < .005). The same pattern was found at T2, with the exception that anchoring, and relationship did not differ between them. Time differences were found for the anchoring dimension, *F*(1, 93) = 10.79, *p* = .001, η^2^_p_ = 0.10, with a decrease in performance from T1 to T2, as well as for the sub thematization dimension *F*(1, 93) = 2.69, *p* < .001, η^2^_p_ = 0.24, with an increase in performance from T1 to T2.
